# Evaluation of novel obesity- and lipid-related indices as predictors of abnormal glucose tolerance in Chinese women with polycystic ovary syndrome

**DOI:** 10.1186/s12902-022-01179-0

**Published:** 2022-11-08

**Authors:** Qianqian Yin, Xiaonan Yan, Yijuan Cao, Jianhua Zheng

**Affiliations:** 1 Center for Reproductive Medicine, XuZhou Central Hospital, No. 199, Jiefang Road, Xuzhou, 221009 Jiangsu China; 2 Department of Obstetrics and Gynecology, XuZhou Central Hospital, No. 199, Jiefang Road, Xuzhou, 221009 Jiangsu China

**Keywords:** Abnormal glucose tolerance, Chinese visceral adiposity index, Visceral adiposity index, Lipid accumulation product, Triglyceride glucose index, Polycystic ovary syndrome

## Abstract

**Purpose:**

We aimed to evaluate the performance of Chinese visceral adiposity index (CVAI), visceral adiposity index (VAI), lipid accumulation product (LAP), triglyceride glucose (TyG) as indices in screening abnormal glucose tolerance (AGT) in Chinese women with polycystic ovary syndrome (PCOS), using the oral glucose tolerance test (OGTT) as a reference test. In addition, we essentially compared the abilities of these indices with body mass index (BMI), waist circumference (WC), fasting plasma glucose (FPG).

**Materials and methods:**

All 1113 PCOS patients evaluated in this study underwent OGTTs. The 2-h post-oral glucose load (2 h-PG) level was used to categorize subjects into two groups: those having AGT or normal glucose tolerance (NGT) levels.

**Results:**

A statistically significant positive correlation between levels of 2 h-PG and FPG, BMI, WC, LAP, VAI, CVAI, TyG, (*P* < 0.05), was observed. The strongest correlation was found between the levels of 2 h-PG and CVAI (r = 0.47). The CVAI provided the highest area under the receiver-operating characteristic curve (AUC) for AGT, followed by LAP, BMI, TyG, VAI, WC, and FPG. The CVAI of 32.61 (with AUC: 0.76, sensitivity: 73%, specificity: 70%, positive preductive value (PPV): 0.41, negative predictive value (NPV): 0.90) was found to be the cut-off point for AGT in Chinese women with PCOS.

**Conclusions:**

CVAI may not reliably detect AGT in Chinese women with PCOS. However, it is suitable as a first screening indicator to guide physicians to ordering OGTT.

## Introduction

Type 2 diabetes mellitus (T2DM), an established risk of cardiovascular disease, disability, and premature death, has become a major worldwide public health burden in the past decade [1, 2]. Impaired glucose tolerance (IGT), at an early stage in the natural history of T2DM, may remain underdiagnosed since it is usually asymptomatic, and its detection necessitates an oral glucose tolerance test (OGTT) [3]. Adoption of suitable lifestyle modifications or pharmacological interventions may delay or prevent deterioration of IGT to T2DM [3]. Therefore, a need for earlier detection of IGT has been significantly highlighted.

Polycystic ovary syndrome (PCOS), a very common endocrine disorder, impacts 5–10% of women in their reproductive age [4]. A more meaningful screening for abnormal glucose tolerance (AGT) is warranted in women with PCOS as they show an increased prevalence of disturbances of glucose metabolism compared with the general population and have shown characteristically postprandial abnormalities in glucose metabolism [5–7].

However, OGTT, considered the gold-standard in detecting AGT, is an inconvenient and time-consuming procedure. Therefore, it should have a simple and effective screening tool for AGT, especially in women with PCOS.

Studies have firmly established that both obesity and dyslipidemia are the traditional risk factors for T2DM because these two conditions can increase peripheral tissue insulin resistance (IR) [8]. Therefore, in general populations of different races, several obesity- and lipid-related indices, including visceral adiposity index (VAI) [9–11], lipid accumulation product (LAP) [9, 12, 13], and triglyceride Glucose index (TyG) [9, 11, 14], have been commonly suggested as promising surrogate indicators of both T2DM and prediabetes. Recently, the Chinese visceral adiposity index (CVAI), a tool developed to estimate visceral obesity of Chinese individuals, has been suggested as a better predictor of T2DM and prediabetes than any other obesity index for Chinese adults [1, 2, 15, 16].

To our knowledge, women with PCOS show more visceral fat accumulation than those without PCOS, even if they are of normal weight, and visceral adiposity is more associated with obesity-related metabolic abnormalities than subcutaneous or peripheral fat accumulation [17]. Therefore, the aforementioned indicators may have different implications for screening of AGT among the PCOS population than in the general population. Moreover, although there are many studies on the relationships of these indices with T2DM and prediabetes, however, the data establishing an association between these indices and AGT remains scarce [8, 12, 18], especially for the PCOS population [19, 20]. Thus, in this cross-sectional study, we aimed to evaluate the diagnostic performance of obesity and lipid-related indices as tools in screening AGT in Chinese women with PCOS, using the OGTT as our reference gold-standard method.

## Material and methods

### Subjects and study design

A total of 1113 women with PCOS (aged 13–41 years) with complete medical records were recruited at the center for reproductive medicine and gynecological outpatient department, Xuzhou Central Hospital (outpatient consultation) from July 2012 to December 2020. Any medications known to affect sex hormone, glucose or lipid metabolism were discontinued for at least three months before the study.

#### PCOS diagnosis

PCOS adults (aged 20–41 years) were diagnosed when meeting at least two out of the following three criteria [4, 7] (PCOS adolescents (aged 13–19 years) required the presence of all three criteria) [21]: (i) olig- and/or anovulation (i.e. eight or fewer menstrual cycles in a year or menstrual cycles more than 35 days in length) (ii) clinical hyperandrogenism (i.e. acne or modified Ferriman–Gallwey scores ≥8) or biochemical hyperandrogenism (i.e. total testosterone (TT) ≥ 2.6 nmol/L, free testosterone (FT) ≥ 20.82 pmol/L); and (iii) polycystic ovaries (i.e. presence of ≥12 follicles in each ovary measuring 2–9 mm in diameter) and exclusion of related disorders (e.g. congenital adrenal hyperplasia, androgen-secreting tumours and Cushing’s syndrome, hyperprolactinemia, thyroid disorders).

#### AGT diagnosis

Glucose tolerance was determined using the following indicators [3]: normal fasting plasma glucose (FPG) (FPG < 6.1 mmol/L); impaired fasting glucose (IFG) = FPG ≥6.1 mmol/L but < 7.0 mmol/L; normal glucose tolerance (NGT) = 2-h post-oral glucose load (2 h-PG) < 7.8 mmol/L; IGT = 2 h-PG ≥7.8 mmol/L but < 11.1 mmol/L; and T2DM = FPG ≥ 7.0 mmol/L or 2 h-PG ≥11.1 mmol/L; prediabetes = IFG or IGT; AGT = 2 h-PG ≥ 7.8 mmol/L.

#### The formulas of indices

Indices were evaluated based on the formulas presented below [7]:

$$\textrm{body}\ \textrm{mass}\ \textrm{index}\ \left(\textrm{BMI}\right)=\textrm{weight}\ \left(\textrm{kg}\right)/\textrm{height}\ {\left(\textrm{m}\right)}^2$$

$$\textrm{LAP}=\left[\textrm{waist}\ \textrm{circumference}\ \left(\textrm{WC}\right)\ \left(\textrm{cm}\right)-58\right]\times \textrm{triglycerides}\ \left(\textrm{TG}\right)\ \left(\textrm{mmol}/\textrm{L}\right)$$

$$\textrm{CVAI}=-187.32+1.71\times \textrm{age}+4.23\times \textrm{BMI}+1.12\times \textrm{WC}\left(\textrm{cm}\right)+39.76\times \log 10\left[\textrm{TG}\ \left(\textrm{mmol}/\textrm{L}\right)\right]-11.66\times \textrm{high}-\textrm{density}\ \textrm{lipoprotein}\ \textrm{cholesterol}\ \left(\textrm{HDL-c}\right)\ \left(\textrm{mmol}/\textrm{L}\right)$$

$$\textrm{VAI}=\left[\textrm{WC}\ \left(\textrm{cm}\right)/\left[36.58+\left(1.89\times \textrm{BMI}\right)\right]\right]\times \left[\textrm{TG}\ \left(\textrm{mmol}/\textrm{L}\right)/0.81\right]\times \left[1.52/\textrm{HDL-c}\left(\textrm{mmol}/\textrm{L}\right)\right]$$

$$\textrm{TyG}=\textrm{Ln}\left[\textrm{fasting}\ \textrm{triglycerides}\ \left(\textrm{mg}/\textrm{dL}\right)\times \textrm{fasting}\ \textrm{glucose}\ \left(\textrm{mg}/\textrm{dL}\right)/2\right]$$

### Demographic information and clinical measurements

All the recruited patients underwent 75-g OGTT and anthropometric measurements. All parameters were measured, as previously described [7, 22].

Fasting blood samples were obtained from PCOS patients between the first and fifth day of menstrual period/withdrawal bleeding. Prolactin and total testosterone (TT) were assessed by chemiluminescence immunometric assay (Beckman Unicel DxI 800). Free testosterone (FT) and thyroid-stimulating hormone were also measured using chemiluminescence immunometric assay (Snibe MAGLUMI 4000; Abbott Immulite 2000 analyzer). 17α-hydroxyprogesterone was measured using the ELISA method. Plasma glucose was measured using glucose oxidase method (Hitachi 7600 autoanalyzer). Plasma insulin was measured using chemiluminescence immunometric assay (Roche e601 analyzer). Total cholesterol (CHOL), triglycerides (TG), high-density lipoprotein cholesterol (HDL-c), and low-density lipoprotein cholesterol (LDL-c) were measured using enzymatic colorimetric method (Hitachi 7600 autoanalyzer).

### Statistical analysis

The data were analyzed by the statistical software SPSS version 24.0 for Windows. We assessed the normality of the distribution of all continuous variables using the Kolmogorov–Smirnov test. As the variables were not normally distributed, continuous variables were described as median with 25th–75th percentile, and the differences between the groups were determined by the Mann–Whitney U test. The association between the 2 h-PG and FPG, obesity-and lipid-related indices were tested by Spearman’s rank correlations analysis. Receiver operating characteristic (ROC) curves and the area under ROC curves (AUCs (95%CI)) were used to assess the sensitivily and secpificity of each index in detecting AGT. The optimal cut-off value of each index was determined through the maximization of the Youden index (sensitivity + specificity − 1). Positive preductive value (PPV) was estimted by true positive cases (TP) and false positive cases (FP), and the formula is PPV = TP / (TP + FP). The negative predictive value (NPV) was assessed by ture negative cases (TN) and fasle negative cases (FN), and the formula is NPV = TN / (TN + FN). Differences in AUCs were assessed by the method described by Hanley and McNeil. *P* ≤ 0.05 (two-tailed) was considered statistically significant.

## Results

In the overall study population, the prevalence of alterations in glucose metabolism was as follows: IFG 39/1113 (3.50%), IGT 202/1113 (18.15%), and T2DM 47/1113 (4.30%).

Of the 47 women with T2DM, 5 were diagnosed based solely on FPG level, 34 on 2 h-PG, and 8 based on composite FPG and 2 h-PG. AGT was observed in 244 women (244/1113, 21.92%), the remaining 869 women (869/1113, 78.08%) demonstrated NGT.

As shown in Table 1, there were higher levels of age, BMI, WC, FPG, FIN, TG, LDL-c, CHOL, FT, LAP, VAI, CVAI, and TyG in subjects with AGT compared to those with NGT, while HDL-c was found lower in the same subjects (*P* < 0.05). No significant difference in TT(*P* > 0.05) was noticed.

**Table 1:** Clinical and laboratory characteristics of PCOS patients with AGT and NGT (median [range])

	NGT(***n*** = 869)	AGT(***n*** = 244)	*P*
Age (years)	26.00 (22.00–29.00)	29.00 (26.00–32.00)	< 0.001
BMI (kg/m^2^)	21.23 (19.22–24.14)	25.41 (22.57–28.60)	< 0.001
WC (cm)	73.00 (68.00–81.00)	84.00 (76.00–92.00)	< 0.001
FPG (mmol/L)	5.00 (4.70–5.30)	5.30 (5.00–5.80)	< 0.001
FIN (uU/ml)	7.10(3.90–11.30)	11.30 (7.45–17.40)	< 0.001
TG (mmol/L)	1.03(0.74–1.38)	1.60(1.05–2.21)	< 0.001
HDL-c (mmol/L)	1.54(1.33–1.80)	1.34(1.16–1.58)	< 0.001
LDL-c (mmol/L)	2.83(2.34–3.35)	3.13(2.59–3.65)	< 0.001
CHOL (mmol/L)	4.75(4.30–5.44)	5.21(4.51–5.73)	< 0.001
LAP	14.96 (7.70–30.40)	41.36(21.80–65.14)	< 0.001
VAI	1.18 (0.78–1.85)	2.17 (1.26–3.48)	< 0.001
CVAI	10.90 (−11.46–40.36)	57.85 (33.90–84.25)	< 0.001
TyG	8.31(7.99–8.64)	8.84(8.39–9.23)	< 0.001
TT (nmol/L)	2.26(1.65–2.90)	2.18(1.62–2.88)	0.189
FT (pmol/L)	10.48 (6.80–16.31)	12.63(8.47–20.23)	0.001

*BMI* body mass index, *WC* waist circumference, *FPG* fasting plasma glucose, *FIN* fasting insulin, *TG* triglycerides, *HDL-c* high-density lipoprotein cholesterol, *LDL-c* low-density lipoprotein cholesterol, *CHOL* total cholesterol, *LAP* lipid accumulation product, *VAI* visceral adiposity index, *CVAI* Chinese visceral adiposity index, *TyG* triglyceride glucose Index, *FT* free testosterone, *TT* total testosterone

Table 2 presents the correlation coefficients between the 2 h-PG and each index. A significant positive correlation between the 2 h-PG and FPG, BMI, WC, LAP, VAI, CVAI, TyG (*P* < 0.05) was observed. The 2 h-PG and CVAI (r = 0.47) revealed the strongest correlation.

**Table 2:** Spearman rank correlations between the 2 h-PG and FPG, obesity-and lipid-related indices

	r	*p*
FPG	0.38	< 0.001
CVAI	0.47	< 0.001
VAI	0.40	< 0.001
LAP	0.44	< 0.001
TyG	0.43	< 0.001
BMI	0.41	< 0.001
WC	0.38	< 0.001

*2 h-PG* 2-h post-oral glucose load, *FPG* fasting plasma glucose, *CVAI* Chinese visceral adiposity index, *VAI* visceral adiposity index, *LAP* lipid accumulation product, *TyG* triglyceride glucose Index, *BMI* body mass index, *WC* waist circumference

**Fig. 1:**
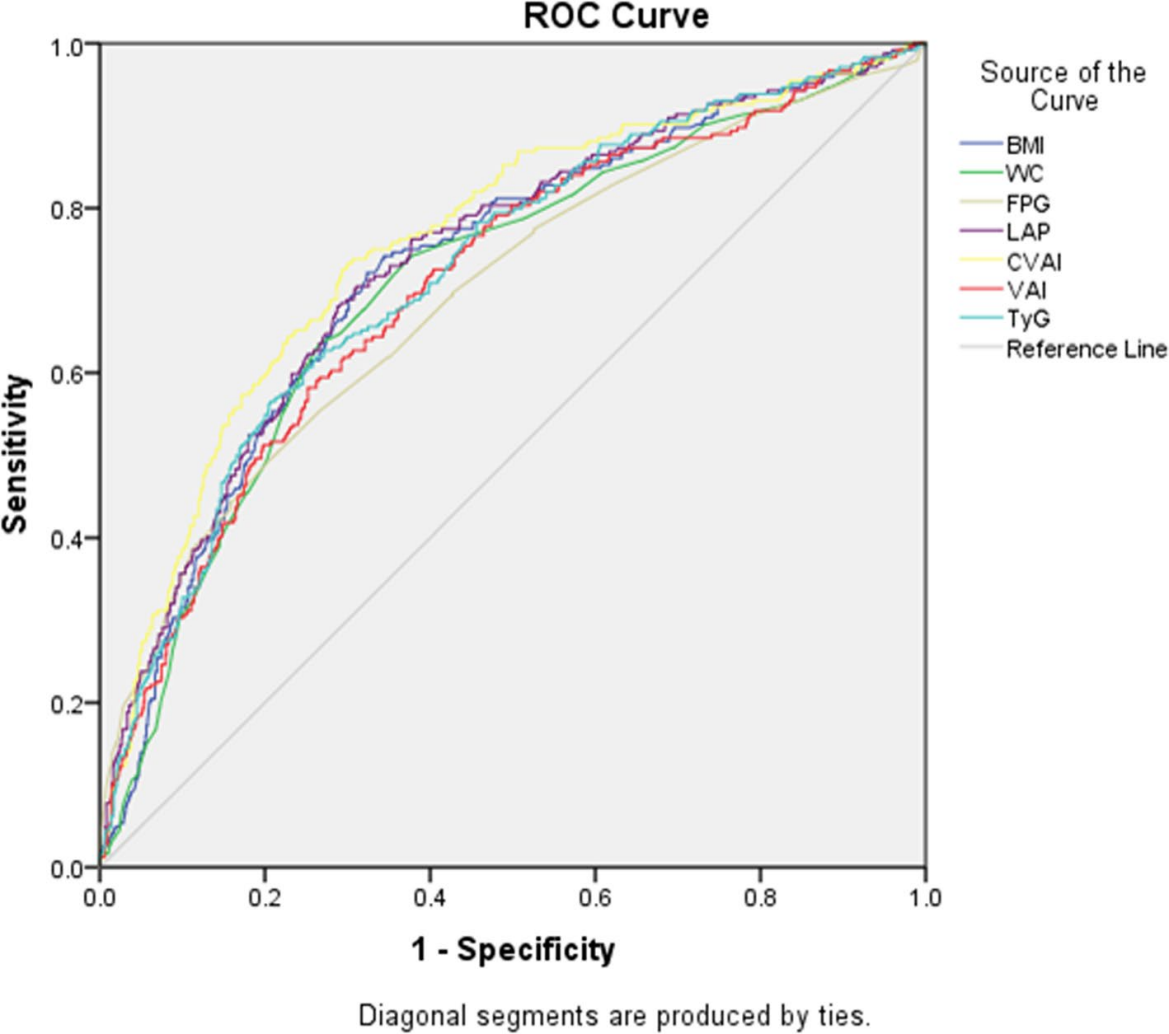
Receiver operating characteristic (ROC) curves for each indicator for detecting abnormal glucose tolerance (AGT) in Chinese women with Polycystic ovary syndrome (PCOS)

In this study, ROC curves analysis indicated that all indices could predict AGT and CVAI, showing the highest AUC, followed by LAP, BMI, TyG, VAI, WC, and FPG. The differences in AUC between CVAI with other indices were statistically significant (*P* < 0.05) (Table 3, Fig. 1).

**Table 3:** ROC curves data for detecting AGT using each indicator

	AUC (95%CI)	Cut-off point	Sen	Spe	Youden index	PPV	NPV	*P*
BMI	0.73(0.69–0.76)	22.70	0.72	0.68	0.40	0.39	0.90	< 0.001
WC	0.71(0.67–0.75)	80.50	0.64	0.73	0.37	0.40	0.88	< 0.001
FPG	0.70(0.66–0.73)	5.35	0.49	0.80	0.29	0.40	0.85	0.006
LAP	0.74(0.70–0.77)	26.19	0.71	0.69	0.40	0.40	0.89	0.004
CVAI	0.76(0.72–0.79)	32.61	0.73	0.70	0.43	0.41	0.90	reference
VAI	0.71(0.67–0.75)	1.85	0.58	0.75	0.33	0.39	0.86	0.001
TyG	0.72(0.69–0.76)	8.71	0.57	0.79	0.36	0.45	0.86	0.037

*P*: CVAI vs other indices

*Sen* Sensitivity, *Spe* Specificity, *PPV* positive preductive value, *NPV* negative predictive value, *BMI* body mass index, *WC* waist circumference, *FPG* fasting plasma glucose, *LAP* lipid accumulation product, *CVAI* Chinese visceral adiposity index, *VAI* visceral adiposity index, *TyG* triglyceride glucose Index

## Discussion

Central obesity and visceral fat are major risk factors for IR. Visceral obesity causes IR by stimulating the formation of matabolic products derived from lipids, hormones, and cytokines [23, 24]. Therefore, various obesity and lipid-related indices have been proposed for detecting IR and prediabetes. In our study, we directly compared the performance of FPG, CVAI, VAI, LAP, TyG, WC, and BMI in detecting AGT and observed FPG demonstrated the lowest AUC value (0.70) and the lowest sensitivity (49%) among all the indices for detecting AGT. Thus, although FPG is an inexpensive assay and does not require mathematical calculations, it may not reliably detect AGT in our PCOS population, a finding consistent with previous studies [3, 5].

We next evaluate the abilities of six obesity- and lipid-related indices (CVAI, VAI, LAP, TyG, BMI, WC) as indicators of detecting AGT in PCOS patients. In line with 2 h-PG as criteria for AGT, our data demonstrated that CVAI outperformed other indices with a higher correlation coefficient and a larger AUC for AGT detection in Chinese women with PCOS.

CVAI, is known as Chinese VAI. VAI, was introduced to estimate visceral adipose function for the Caucasians and is an index established with the use of BMI, WC, TG, and HDL-c [10, 25]. In Caucasians, VAI was used as a valuable indicator of visceral adiposity and adipose tissue dysfunction to predict the risk for cardiovascular diseases and insulin resistance [25]. However, a previous study showed that VAI was poorly associated with adipose tissue area and has poor diagnostic performance to predict prediabetes and diabetes in Chinese [16]. In conformity with previous findings, we showed that the AUC of VAI for AGT was 0.71, which was lower than the AUC of CVAI (0.76), LAP (0.74), TyG (0.72), and BMI (0.73). Moreover, data based on correlation analyses also revealed similar tendencies. The main reason of the discrepancy may be ethnic difference and subject characteristic. VAI was developed initially for Caucasians. However, compared to equivalent Caucasians, Asian subjects have a greater proportion of body fat for a given BMI level [26] and are more prone to accumulate fat around the abdomen [27].

The CVAI index based on the combination of age and all the parameters of VAI (BMI, WC, TG and HDL-c) was developed to estimate visceral fat area for Chinese by Xia and colleagues [16]. In their study, among 6495 middle-aged and elderly Chinese, CVAI provided higher AUCs for the diagnosis of T2DM and prediabetes than BMI, WC and VAI [16]. Also, two prospective studies indicated that CVAI proved a better predictor of prediabetes and diabetes compared to BMI, WC, and VAI in the general Chinese population [15, 28]. However, no data are currently available establishing any association between CVAI and AGT. In our cohort of PCOS patients, CVAI provided the strongest correlation with 2 h-PG and the largest AUC for detecting AGT, thereby suggesting that CVAI might be a more useful indicator of AGT than LAP, TyG, BMI, VAI, and WC in Chinese women with PCOS. Although the calculation of CVAI requires more parameters and more complicated formulas, the parameters (age, BMI, WC, TG, and HDL-c) are easily available and low cost in clinical practice, and the complex algorithms can easily be performed by Tablets or Smartphone. In our study population, ROC curves analysis suggested a threshold value of 32.61 in CVAI for the detection of AGT (73% sensitivity, 70% specificity, 0.76 AUC, 0.41 PPV, 0.90 NPV).

LAP, a novel index based on a combination of WC and TG, was first introduced for the U.S. National Health and Nutrition Examination Survey [29]. Furthermore, it is proposed as a valuable marker indicating T2DM and prediabetes [9, 12]. However, few studies have explored the association of LAP with AGT. The results of previous studies indicated that LAP performed better than BMI in identifying abnormal glucose regulation in the young Korean women and provided higher AUC in estimating the risk for IGT than BMI and waist-to-hip ratio in Austrian PCOS women [12, 19], which was accordance with our results. However, these studies did not compare the utility of LAP with other novel obesity- and lipid-related indices. Our analysis showed that LAP performed superior to TyG, VAI, BMI, and WC, but inferior to CVAI in detecting AGT in Chinese PCOS population. According to the maximized Youden index, the LAP 26.19 (with AUC 0.74, sensitivity 71%, specificity 69%) was found to be the cut-off point for AGT in our study population, which was much higher than that in Korean PCOS women (12.98) [20]. This discrepancy could be partially due to ethnic difference.

TyG index, a composite indicator composed of TG and FPG, is a key predictor of T2DM and prediabetes in severl epidemiological studies. The TyG performed better than homeostasis model assessment of insulin resistance (HOMA-IR) and TG/HDL-c in predicting T2DM in Korean adults without initial T2DM [30]. A study conducted among 3307 elderly Colombian individuals suggested better discriminative power of the TyG index than BMI, WC, and VAI to predict prediabetes [11]. However, OGTT was not performed, and prediabetes was defined as a FPG of 100 to 125 mg/dL (5.6 to 7.0 mmol/L) in this study. In a prospective cohort study conducted on 4543 Chinese individuals without initial prediabetes or diabetes, Wen et al. reported that TyG provided a larger AUC for predicting prediabetes and isolated IGT than FPG, WC, and BMI [8]. However, this study did not compare the ability of TyG with other novel obesity- and lipid-related indices. In the current study, TyG was found inferior to CVAI and LAP for detecting AGT based on AUC and correlation coefficient.

Due to the fact that a plasma insulin assay is not yet available in all laboratories, has poor reproducibility and is costly [31], we do not evaluate the performance of the indices those using insulin for detecting AGT. Indeed, they do not perform better than obesity- and lipid-related indices studied in our report (data not shown).

Despite these relevant findings, it is important to point out the limitation of our study. All the subjects were recruited from Xuzhou Central Hospital, and only those with complete medical records. This population may not represent all the Chinese women with PCOS, with a possibility of bias in the results.

In conclusion, CVAI performed better than LAP, TyG, VAI, BMI, WC, and FPG for detecting AGT in Chinese women with PCOS. Due to the lower sensitivity and specificity, CVAI may not reliably detect AGT in our PCOS cohort. However, because of its good NPV, it is suitable as a first screening indicator to guide physicians to ordering OGTT. Furthermore, treatment plans (metformin or intense lifestyle interventions) could be used to prevent or slow the development of AGT in the women with elevated CVAI.

## Data Availability

The data used to support the findings of this study are available from the corresponding author upon request.

## Abbreviations

T2DM: type 2 diabetes mellitus
IGT: impaired glucose tolerance
OGTT: oral glucose tolerance test
PCOS: polycystic ovary syndrome
AGT: abnormal glucose tolerance
IR: insulin resistance
VAI: visceral adiposity index
LAP: lipid accumulation product
TyG: triglyceride Glucose index
CVAI: Chinese visceral adiposity index
TT: total testosterone
FT: free testosterone
FPG: fasting plasma glucose
IFG: impaired fasting glucose
NGT: normal glucose tolerance
2 h-PG: 2-h post-oral glucose load
CHOL: total cholesterol
TG: triglycerides
HDL-c: high-density lipoprotein cholesterol
LDL-c: low-density lipoprotein cholesterol
BMI: body mass index
ROC: Receiver operating characteristic
AUCs: ROC curves
PPV: positive preductive value
TP: true positive cases
FP: false positive cases
NPV: negative predictive value
TN: ture negative cases
FN: fasle negative cases
HOMA-IR: homeostasis model assessment of insulin resistance
